# Application of Fluorescent Carbon Dots as Catalysts for the Ring-Opening Reaction of Epoxides

**DOI:** 10.3390/ma16247620

**Published:** 2023-12-13

**Authors:** Diana M. A. Crista, Joaquim C. G. Esteves da Silva, Luís Pinto da Silva

**Affiliations:** 1 Chemistry Research Unit (CIQUP), Institute of Molecular Sciences (IMS), Department of Geosciences, Environment and Territorial Planning, Faculty of Sciences, University of Porto, R. Campo Alegre s/n, 4169-007 Porto, Portugal; up200702319@fc.up.pt (D.M.A.C.);; 2 LACOMEPHI, GreenUPorto, Department of Geosciences, Environment and Territorial Planning, Faculty of Sciences, University of Porto, R. Campo Alegre s/n, 4169-007 Porto, Portugal

**Keywords:** carbon dots, organocatalysts, hydrogen bond donor, epoxides, CO_2_ conversion

## Abstract

Considering the increased anthropogenic emissions of CO_2_ into the atmosphere, it is important to develop economic incentives for the use of CO_2_ capture methodologies. The conversion of CO_2_ into heterocyclic carbonates shows significant potential. However, there is a need for suitable organocatalysts to reach the required efficiency for these reactions. Given this, there has been an increasing focus on the development of organocatalytic systems consisting of a nucleophile and a hydrogen bond donor (HBD) so that CO_2_ conversion can occur in ambient conditions. In this work, we evaluated the potential of fluorescent carbon dots (CDs) as catalytic HBDs in the ring-opening reaction of epoxides, which is typically the rate-limiting step of CO_2_ conversion reactions into heterocyclic carbonates. The obtained results demonstrated that the CDs had a relevant catalytic effect on the studied model reaction, with a rate constant of 0.2361 ± 0.008 h^−1^, a percentage of reactant conversion of 70.8%, and a rate constant enhancement of 32.2%. These results were better than the studied alternative molecular HBDs. Thus, this study demonstrated that CDs have the potential to be used as HBDs and employed in organocatalyzed CO_2_ conversion into value-added products.

## 1. Introduction

The development of the world economy and industry has led to an unprecedented increase in anthropogenic carbon dioxide (CO_2_) emissions into the atmosphere. This had a significant impact on the ecological environment of the earth, with consequences such as glacier melting, the rise of the sea level, and Earth’s average surface temperature being correlated to the increased CO_2_ levels [1,2,3]. Despite its harmful consequences when mismanaged, this gas can also be regarded as an environmentally friendly, abundant, and low-cost C1 source [4,5,6,7,8]. Given this, the utilization of excess CO_2_ as a carbon feedstock in organic synthesis has become an important goal for researchers. More specifically, it may provide economic incentives for the development and employment of carbon capture strategies.

The development of novel catalytic approaches and innovative pathways for CO_2_ fixation are being developed continuously [9]. One of the routes for CO_2_ conversion into value-added products occurs via the combination of CO_2_ with epoxides to produce cyclic organic carbonates [10,11,12,13]. The advantage of this pathway is that the production of heterocyclic carbonates through CO_2_ epoxide coupling is an environmentally friendly and toxicological alternative to conventional methods involving diols and toxic phosgenes [14,15,16]. Heterocyclic carbonates are an important and versatile class of chemicals that have several applications, such as electrolytes for lithium-ion batteries [17], in the synthesis of polymers (as polycarbonates) [18], pharmaceutical manufacturing [19], and polar aprotic solvents [20,21,22].

However, due to the thermodynamic stability and kinetic inertia of CO_2_, there are restrictions in terms of the efficiency of the CO_2_ fixation/conversion processes. Thus, there is a need for novel and effective catalytic systems that can overcome these limitations and improve the efficiency of CO_2_ conversion processes [23].

Over the years, the focus of the scientific community in terms of CO_2_ conversion into heterocyclic carbonates has been the development of both transition metal-based and organo-based approaches [24,25]. Transition metal-based catalysts have shown promising results by allowing the CO_2_ conversion reactions to proceed with good yields at ambient temperatures. However, they also show some relevant disadvantages, such as requiring harsh preparation conditions, presenting low thermal stability, and higher prices. On the other hand, organocatalysts are generally low cost, nontoxic, and stable compounds that show inertness toward moisture and air. In some cases, they can be obtained from renewable feedstocks, benefiting these processes in terms of environmental sustainability. So, it is not surprising that different researchers have been interested in using organocatalysts in CO_2_ conversion reactions [9,26,27,28,29]. Nevertheless, organocatalytic systems also present some limitations, such as requiring higher temperatures and CO_2_ pressures [9]. To overcome these issues, researchers have been focused on the development of two-component organocatalytic systems, which are composed of a nucleophile and a hydrogen bond donor (HBD), in which the limitations of both transition metal-based catalysts and organocatalysts can be bridged [30,31,32,33].

**Scheme 1:**
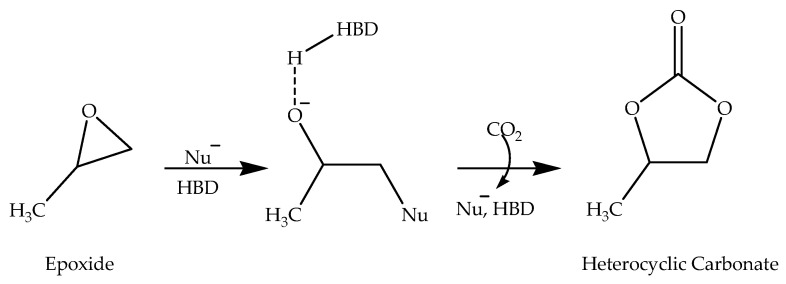
Reaction scheme for CO_2_ conversion in the presence of a nucleophile (Nu^−^) and a hydrogen bond donor (HBD).

In this type of system, with the goal of facilitating the epoxide’s ring opening by nucleophilic attack, the HBDs interact with the O-donor of the epoxide via hydrogen bonding in a process involving a suitable nucleophile (Scheme 1). Furthermore, the HBDs are expected to stabilize the oxyanion resulting from the ring-opening reaction and via hydrogen bonding. These synergistic effects should make the ring-opening reaction of the epoxide more efficient, allowing the production of heterocyclic carbonates from CO_2_ at lower temperatures and CO_2_ pressures [13,18,34,35,36]. It should be noted that in this type of catalytic scheme, the ring opening of the epoxide through nucleophile attack typically represents the rate-limiting step of the process [13,14,17,37,38]. So, the addition of an HBD is made with the expectancy of decreasing the activation barrier for this step, thus increasing efficiency [39]. Considering this, there is a growing interest in the development and identification of new HBDs with the potential to be used in CO_2_ conversion reactions.

Carbon dots (CDs) are a class of fluorescent carbon-based nanoparticles with a quasi-spherical shape and a size usually below 10 nm [40,41,42]. These nanoparticles possess remarkable properties such as high photoluminescence [43,44,45,46], low toxicity, biocompatibility [47,48], high photostability and chemical stability, and good water solubility [40,43]. Thus, CDs have gained relevance in various fields, such as sensing, bioimaging, drug delivery, photodynamic therapy, and photocatalysis. Depending on the used precursors and synthesis conditions, CDs may present distinct properties, compositions, and potential applications. They are often synthesized from carbon-rich precursors and heteroatom-doping molecules, which can include organic compounds containing oxygen, nitrogen, and other heteroatoms [40,41,42,49]. Considering that hydroxyl and amino groups are commonly found on the surface of CDs and that they can participate in hydrogen bonding with acceptor molecules, the presence of these oxygen- and nitrogen-based groups on the CD surface allows them to act as HBDs [50]. As so, CDs could potentially be employed as an HBD for the synthesis of cyclic organic carbonates from CO_2_ and epoxides.

In this work, we will evaluate the potential application of CDs, a low-cost and easy-to-prepare nanomaterial, as HBDs in two-component organocatalytic systems for CO_2_ fixation at room temperature, without the need for high temperatures and pressure conditions, which should be an advantage when compared with current organocatalysts. These CDs result from the dry heating of solid mixtures of glucose (carbon precursor) and urea (N-dopant). Here, we will specifically focus on the rate-limiting step of the process, namely the epoxide’s ring-opening reaction in which the HBD should act. To this end, we tested the use of CDs as HBDs in a model reaction using propylene oxide as the epoxide and pyridine as a nucleophile. Pyridine was chosen due to the fact that after catalyzing the ring opening, it traps the intermediate species of the reaction, allowing a better study of this reaction step [39]. To ascertain the benefits of our method, the results obtained with the CDs were compared with other potential HBDs. More specifically, given that the CDs were prepared from glucose, we compared them with glucose itself and two other saccharides (fructose and sucrose). The CDs showed superior catalytic activity, thereby demonstrating both the advantages of converting glucose into CDs for this application and the better activity of CDs compared with alternative molecular HBDs.

## 2. Materials and Methods

### 2.1. Reagents

The following reagents were used as received: propylene oxide 99% (Sigma, St. Louis, MO, USA), pyridine ACS Reag. Ph Eur (Merck, Darmstadt, Germany), D-(+)-glucose ≥ 96% (Sigma, St. Louis, MO, USA), D(−)-fructose ≥ 98% (Panreac, Darmstadt, Germany), sucrose (Sigma, St. Louis, MO, USA), and urea (Sigma, St. Louis, MO, USA).

### 2.2. Experimental Details

#### 2.2.1. Synthesis of CDs

The CDs were synthesized following a methodology previously developed by us [50]. Briefly, CDs were prepared via a one-pot solvent-free dry-heating method, using glucose and urea as precursors in a molar ratio of 1:1. The solid precursors were placed in a Teflon-lined reactor encased in a stainless-steel shell and heated in an oven for 2 h at 200 °C. The synthesized nanoparticles were purified by centrifugation (10 min/12,000 rpm) and dialysis (Float-A-Lyzer^®^ G2 Dyalisis Device, Sigma, St. Louis, MO, USA, MWCO:500 Da, 24 h). The CDs were characterized in our previous work by high-resolution transmission electron microscopy (HR-TEM), X-ray photoelectron (XPS), fluorescence, and UV-Vis spectroscopy, as well as by assessing the thermal stability of the nanoparticles [50].

#### 2.2.2. Monitoring of the Ring-Opening Reaction

The reaction mixture was prepared by adding propylene oxide (2 mmol) and pyridine (10% mmol) in 5 mL of deionized water. The HBD was added to the reaction mixture in a concentration ranging between 5 and 100 µg/mL [34]. After preparation, the mixtures were placed in a water bath (25 °C, stirring rate of 500 rpm). Kinetic studies were performed by measuring the pyridine peak area via HPLC-DAD for reaction times of 0, 0.5, 1, 2, 3, 4, and 5 h. Each sample was made in triplicate and diluted 200 times in the correspondent mobile phase.

#### 2.2.3. Chromatographic Conditions

The chromatographic system consisted of an isocratic pump (Hewlett-Packard 1100 Series, Boeblingen, Germany), a manual sample injection valve with a 20 µL loop (Rheodyne 7725i, Rohnert Park, CA, USA), a silica-based C18 reversed-phase column (Thermo-scientific Acclaim™ 120 column 4.6 × 150 mm, particle size 3 µm, pore diameter 120 Å), and a photodiode array detector (UV 6000LP, Thermo Scientific, San Jose, CA, USA). The mobile phase was composed of methanol and water (80:20% *v*/*v*). Chromatographic assays were performed at a constant flow rate (0.5 mL min^−1^) under isocratic conditions. Absorbance was monitored in a total scan mode from 210 to 600 nm. The system was controlled by Xcalibur version 1.4 SR software.

### 2.3. Fluorescence Measurement

Fluorescence analysis was performed using a Horiba Jobin Yvon Fluoromax-4 spectrofluorometer (Madrid, Spain) and standard 10 nm fluorescence quartz cells. Fluorescence spectra were obtained with a 1 nm interval and 5 nm slit widths. The samples were analyzed in an aqueous solution with 2 mmol of propylene oxide, 10% mmol of pyridine, and 12.5 µg/mL of CDs for reaction times of 0, 0.5, 1, 2, 3, 4, and 5 h.

### 2.4. Data Analysis

Monitoring the conversion of pyridine as a function of the time of reaction was carried out via HPLC analysis. The kinetic curves gave rise to rate constants (*k*, in h^−1^). The percentage of pyridine conversion (%PC), the *k*^NC^ without a catalyst and *k*^C^ with a catalyst, and the percentage of rate constant enhancement when the HBD was present (%Inc) were calculated using Equations (1)–(3) [51], respectively:

%PC = 100 × (A_0_ − A_5h_)/A_0_, (1)

ln (A_t_/A_0_) = −*kt*, (2)

%Inc = 100 × (*k*^C^ − *k*^NC^)/*k*^NC^, (3)

where A_0_ and A_t_ are the pyridine peak integrated area at the start and *t* time of the reaction, respectively. The pyridine peak was measured since the HPLC method was defined based on this compound.

## 3. Results and Discussion

In this study, we aimed to evaluate the potential of CDs as an HBD in a two-component organocatalytic system for the ring-opening reaction of a model epoxide, propylene oxide. This is typically considered the rate-limiting step in the CO_2_ conversion into value-added heterocyclic carbonates. Thus, an enhanced catalytic activity in this reaction step is required for the organocatalyzed CO_2_ conversion to occur at lower temperatures.

**Scheme 2:**
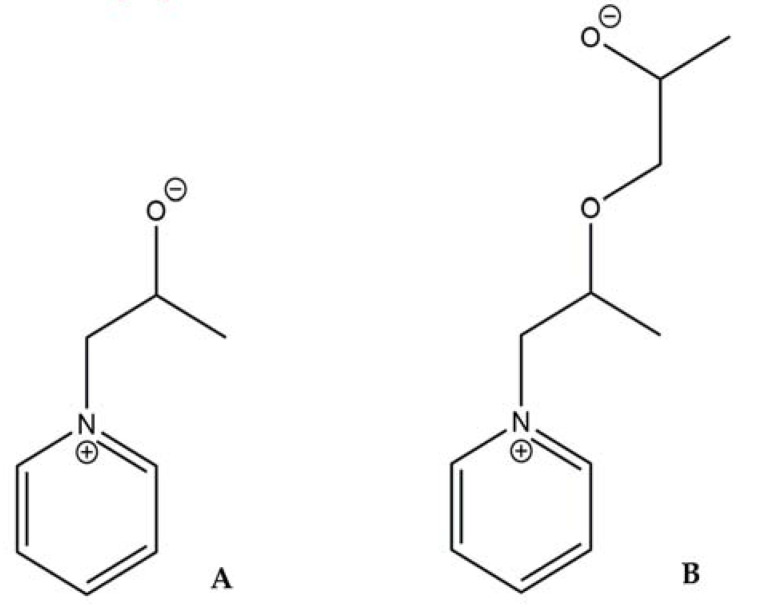
Possible complexes formed between pyridine and propylene oxide. Complex A is formed between one pyridine and one propylene oxide molecule. Complex (**B**) is formed between Complex (**A**) and another propylene oxide molecule [39].

The herein-studied CDs were synthesized and characterized in a previous work by our team [50]. Briefly, they were fabricated via the dry heating of solid mixtures of glucose (as carbon precursor) and urea (as the N-dopant) in a 1:1 molar ratio. The CDs were then incorporated as HBDs in a catalytic system in conjugation with pyridine in a two-component organocatalytic system. We used pyridine as a nucleophile due to previously observing that pyridine forms two types of stable complexes with ring-opened epoxides, allowing us to trap the resulting oxyanion intermediate and monitor its formation [39]. A single pyridine molecule can be involved in the ring-opening reaction of up to two molecules of propylene oxide (Scheme 2). More specifically, a complex formed by one pyridine and one propylene oxide is formed via a nucleophilic attack by the former, originating the expected oxyanion. Subsequently, the deprotonated oxygen heteroatom can attack another epoxide molecule, with the formation of an ether bond. The second complex is expected to be formed in a higher ratio [39]. The formation of these complexes was previously investigated by UV-Vis spectroscopy and mass spectrometry, with density functional theory (DFT)-based calculations assisting in the characterization of the postulated reaction mechanism [39].

It is known that water can act as an HBD, with similar results to other molecules [39]. In order to test if using CDs as HBDs instead of water results in any relative enhancement of the catalytic activity, the catalytic system will be tested in an aqueous solution.

**Figure 1:**
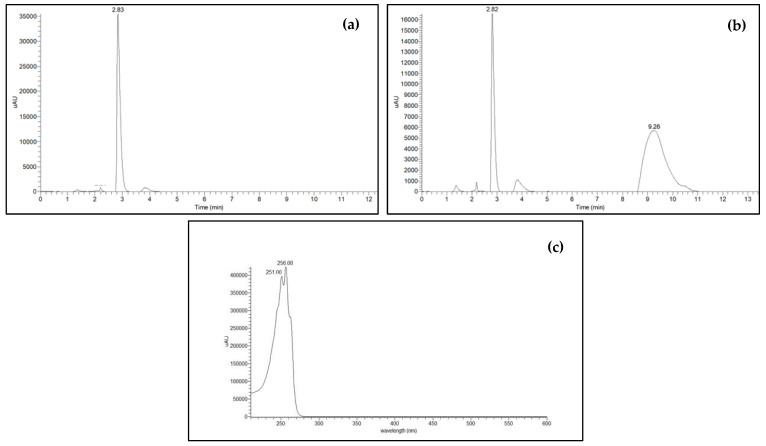
Chromatogram for (**a**) T_0_ and (**b**) T_5h_ for the reaction in the absence of CDs and (**c**) UV-Vis spectrum for a peak at ~2.8 min at T_0_.

The ring-opening reaction of propylene oxide was evaluated using HPLC-DAD via monitoring the consumption of pyridine for the formation of the oxyanion complexes. We started by measuring this ring-opening reaction using a mixture of pyridine and propylene oxide in an aqueous solution and in the absence of CDs (Figure 1). We can see that before the reaction (t_0_), there is a peak with a retention time of 2.8 min, which is ascribed to pyridine. This is confirmed by the corresponding UV-Vis spectrum, which is presented in Figure 1c and matches the absorption profile of pyridine. Figure 1b presents the chromatogram obtained after five hours of reaction (t_5h_). Here, we can observe the formation of a new peak with a retention time of 9.2 min and a corresponding decrease in the peak associated with pyridine. The appearance of a new product with the consumption of pyridine is expected when we consider the use of the latter as a nucleophile in the ring-opening reaction of epoxides [39]. Given this, we attributed the new peak to product complexes formed between pyridine and propylene oxide. It should be noted that this new peak is not so well defined, which might be explained by the fact that these chromatographic conditions were optimized for pyridine, and so it might not be able to properly elute or separate the two potential product complexes. Nevertheless, subsequent kinetic measurements will be made by considering the pyridine peak alone, which is not affected by the peak ascribed to the products.

The next step was to evaluate the potential HBD properties of the CDs in this reaction. To that end, we analyzed the reaction mixture with increasing concentrations of CDs: 5 µg/mL, 12.5 µg/mL, 25 µg/mL, 40 µg/mL, 50 µg/mL, and 100 µg/mL.

**Figure 2:**
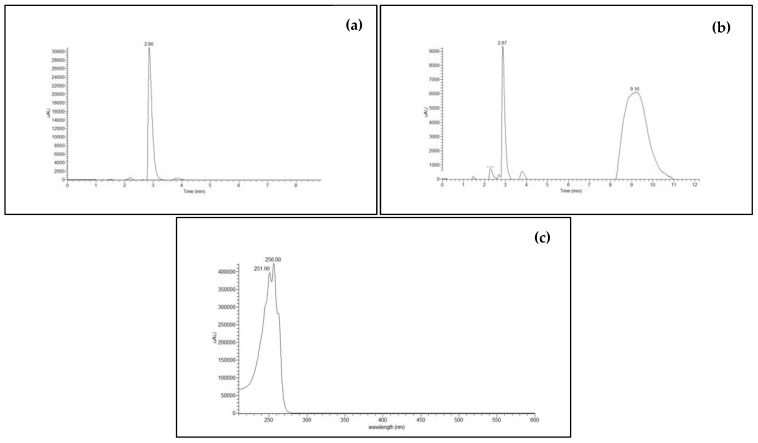
Chromatogram for (**a**) T_0_ and (**b**) T_5h_ for the reaction with CDs and (**c**) UV-Vis spectrum for a peak at ~2.8 min at T_0_.

Figure 2a presents the chromatogram obtained at t_0_ for the reaction in the presence of CDs (12.5 µg/mL), while Figure 2b corresponds to the chromatogram obtained at t_5h_. These results indicate that there are no qualitative differences between the reactions with and without CDs. We still observe a pyridine peak at 2.87 min and a peak attributed to the pyridine–propylene oxide complex at 9.16 min. There is also no difference regarding the UV-Vis spectrum corresponding to the pyridine peak (Figure 1c). However, with the addition of CDs, when compared to the reaction in their absence, we observed a decrease in the area of the pyridine’s peak with a corresponding increase in the area ascribed to the products’ peak. Thus, the addition of CDs led to quantitative differences.

**Figure 3:**
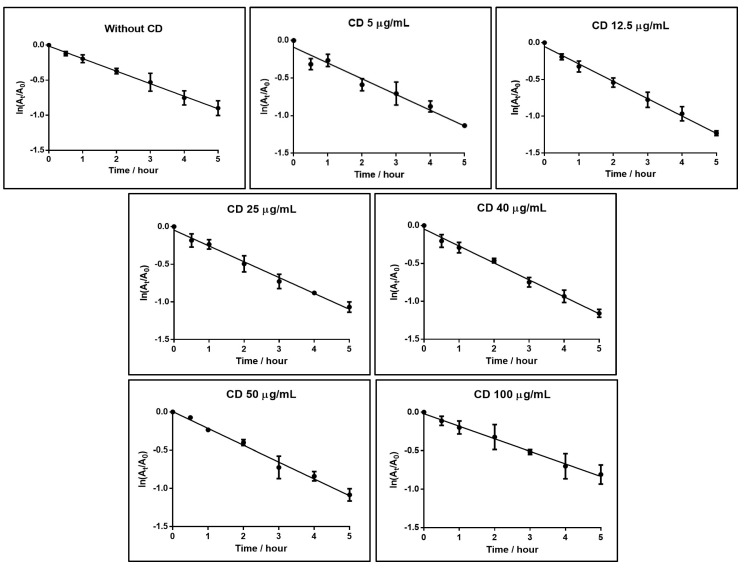
Linear fitting of ln(A_t_/A_0_) as a function of time (in hours) for the reaction mixtures composed of propylene oxide, pyridine, and CDs in aqueous solution.

The kinetics of these reactions, with increasing concentrations of CDs, were determined by correlating the absolute area of the pyridine’s peak in the HPLC chromatogram with the different reaction times. Rate constants (*k*, in h^−1^) were obtained for each concentration of CDs (µg/mL). These values resulted from a linear fitting. Figure 3 represents the linear fitting for all the analyzed concentrations.

The rate constant for each CD concentration is presented in Table 1, as well as the %Inc relative to the reaction without CDs, and %PC. When comparing the results in the presence and absence of the CDs, we can observe that adding this type of nanomaterial led to an overall increase in the reaction rate constant. The exception is for the highest concentration of CDs, which can potentially be justified by taking into account their typical aggregation behavior (associated with the typical surface composition of these materials [52,53]). Namely, by the presence of typical carboxyl, amine, and carbonyl groups, with the subsequent formation of electrostatic interactions among nanoparticles, as well as hydrogen bonding. Thus, it is possible that at higher concentrations, this aggregation occurs in a way that might limit the ability of CDs to act as HBDs in this reaction, resulting in lower catalytic activity. On the other hand, a 5 µg/mL concentration of CDs resulted in a catalytic activity lower than that obtained with 12.5 µg/mL of CDs. This could simply be because the lower concentration of CDs is not enough to yield a good enough catalytic activity. Finally, the correlation between the CD concentration and the increased efficiency is not linear. In fact, the concentration of 12.5 µg/mL yielded the best results, presenting a rate constant of 0.2361 ± 0.008 h^−1^, a %PC of 70.8%, and a %Inc of 32.2%, whereas the second-best results were not obtained with either the first or third lowest concentration, as would be expected if the correlation was linear.

**Figure 4:**
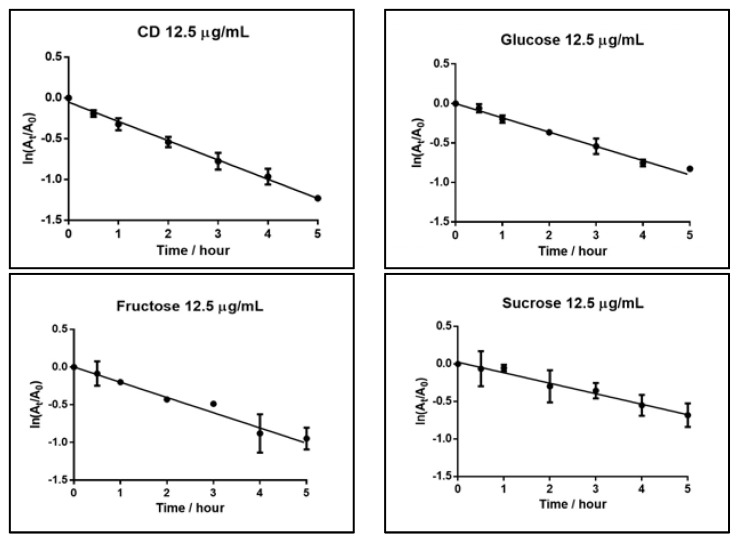
Linear fitting of ln(A_t_/A_0_) as a function of time (in hours) for the reaction mixtures composed of propylene oxide, pyridine, and HBD (CDs, glucose, fructose, and sucrose) in aqueous solution.

Based on these results, it is clear that the CDs can act as an HBD in this type of reaction and provide better results over water molecules than HBDs. These results are in line with our previous work [50], since by XPS analysis it was found that the surface of CDs contained functional groups, such as hydroxyl and amine ones, which can act as HBDs. Considering this, we have decided to compare the enhancement effect obtained with the CDs with what could be achieved using other potential HBDs. To that end, we compared the catalytic activity of the CDs with three saccharides: glucose, fructose, and sucrose. These HBDs were chosen due to representing the type of carbon source used in the CDs and presenting functional groups capable of hydrogen bonding. In fact, saccharides were already used as catalysts in the cycloaddition of CO_2_ to epoxides [22,32,39]. With this comparison, we aim to understand if the conversion of glucose in CDs results in a better catalytic activity than the direct use of glucose (and the other saccharides) as HBDs. Figure 4 presents the linear fitting of ln (A_t_/A_0_) as a function of time in the presence of the three saccharides and the optimal tested concentration of CDs (12.5 µg/mL).

The rate constant, the %Inc, and %PC obtained for each fitting are present in Table 2.

The %Inc was calculated using the results obtained in the absence of CDs as the reference. The results indicate that the use of CDs promotes a better catalytic activity than the use of the saccharides by themselves. Using fructose, the best-performing saccharide, we obtained a %Inc of 13.0 and a %PC of 66.9, which are worse results than what was obtained using CDs but better than those obtained when just using water as an HBD. For glucose, the %Inc was 1.0, which is almost the same result obtained in the absence of CDs. Finally, for sucrose, the %Inc was −21.7 and the %PC was 49.1, indicating that using this saccharide instead of using only water actively worsens the catalytic performance. From these results, we can conclude that the conversion of glucose into CDs provides benefits in terms of catalytic activity, with the CDs presenting an enhanced catalytic activity compared to the use of other potential HBDs.

**Figure 5:**
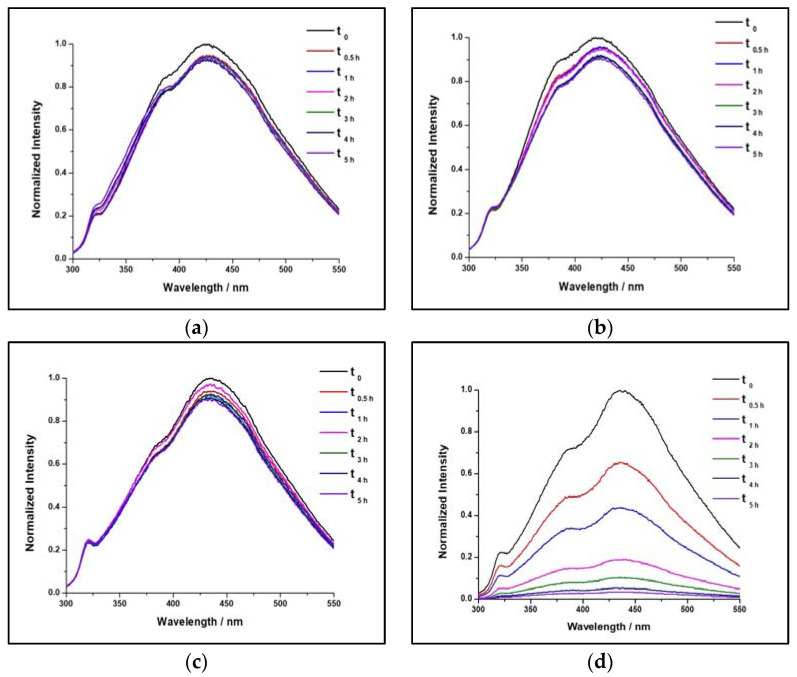
Emission spectra of (**a**) CDs in water; (**b**) CDs in water with 2 mmol of propylene oxide; (**c**) CDs in water with 10% mmol of pyridine; (**d**) complete reaction mixture during a 5 h reaction.

Finally, we measured how the fluorescence of the CDs is affected by the ring-opening reaction of propylene oxide in aqueous solution. All the emission spectra were measured using an excitation wavelength of 290 nm. The fluorescence intensity was analyzed in four different scenarios: CDs in aqueous solution; CDs in aqueous solution with propylene oxide; CDs in aqueous solution with pyridine; CDs in aqueous solution with propylene oxide and pyridine (ring-opening reaction). The results are presented in Figure 5. As we can observe, the different components of the ring-opening reaction mixture do not affect the emission wavelength maximum (~460 nm) of the CDs over the course of the 5 h reaction period. Interestingly, in the presence of each individual component, the variation of the CD fluorescence intensity is quite limited. However, when in the presence of all of the components, we can observe a significant quenching of the fluorescence, indicating that the CDs interact with compounds generated during the ring-opening reaction. These further cements the participation of the CDs in the reaction to the role of an HBD.

## 4. Conclusions

In this work, we have evaluated the potential of CDs to act as HBDs in two-component organocatalytic systems for the ring-opening reaction of epoxides (which can be considered a rate-limiting step in CO_2_ conversion into heterocyclic carbonates). The CDs were prepared via dry heating of solid mixtures of glucose and urea, as a carbon precursor and an N-dopant (respectively). The CDs were tested using a model system composed of propylene oxide (used as a model epoxide) and pyridine (used as the nucleophile). The reactions that employed CDs, a low-cost and easy-to-prepare nanomaterial, took place in aqueous solution at room temperature. Pyridine was selected as a nucleophile, as its use generates a ring-opened epoxide-nucleophile structure that is stable enough for this ring-opening reaction to be properly monitored.

Our results demonstrated that CDs possess a significant catalytic activity that results in a substantial increase in reaction efficiency compared to the use of water and other molecules (e.g., saccharides) for the same purpose. More specifically, CDs showed a relevant catalytic performance, with a rate constant of 0.2361 ± 0.008 h^−1^, a percentage of reactant conversion of 70.8%, and a rate constant enhancement of 32.2%. A comparison with alternative molecular HBDs (glucose, fructose, and sucrose) showed the superior performance of the studied nanomaterials and the benefits of converting glucose (carbon precursor) into CDs. This work demonstrated that CDs possess catalytic activity in the ring-opening reaction of epoxides, which shows their potential to be used as HBDs in the catalytic conversion of CO_2_ into heterocyclic carbonates. Therefore, this work provides a basis on which future works concerning the incorporation of CO_2_ into value-added products can be built.

## Figures and Tables

**Table 1:** Rate constant (*k*, h^−1^), %Inc, and %PC for the reactions in the presence of different concentrations of CDs. All the samples were analyzed in triplicate. The fitting of catalytic reactions resulted in R^2^ > 0.98.

	*k* (h^−1^)	%Inc	%PC
Without CD	0.1786 ± 0.009		59.1
CDs 5 µg/mL	0.2093 ± 0.014	17.2	67.8
CDs 12.5 µg/mL	0.2361 ± 0.008	32.2	70.8
CDs 25 µg/mL	0.2101 ± 0.010	17.6	65.6
CDs 40 µg/mL	0.2233 ± 0.008	25.0	68.6
CDs 50 µg/mL	0.2201 ± 0.010	22.3	66.1
CDs 100 µg/mL	0.1629 ± 0.012	-8.8	55.2

**Table 2:** Rate constant (*k*, h^−1^), %Inc, and %PC, for the reactions with HBD (CDs, glucose, fructose, and sucrose). All the samples were analyzed in triplicate. The fitting of catalytic reactions resulted in R^2^ > 0.98.

	−*k* (h^−1^)	%INC	%PC
CD 12.5 µg/mL	0.2361 ± 0.008	32.2	70.8
Glucose	0.1804 ± 0.008	1.0	56.2
Fructose	0.2019 ± 0.018	13.0	66.9
Sucrose	0.1399 ± 0.016	−21.7	49.1
